# Cold Stress Responses of Different Genotypes of *Miscanthus* Assessed by Relative Electrical Conductivity and LT_50_

**DOI:** 10.3390/plants14121760

**Published:** 2025-06-09

**Authors:** Ning Peng, Songtao Guo, Yanmei Tang, Shicheng Li, Trang Pham, Xianyan Kuang, Zili Yi, Liang Xiao

**Affiliations:** 1 College of Bioscience & Biotechnology, Hunan Agricultural University, Changsha 410128, China; pengning28@stu.hunau.edu.cn (N.P.); gst0208@163.com (S.G.); tangyanmei@stu.hunau.edu.com (Y.T.); lishicheng@stu.hunau.edu.cn (S.L.); yizili889@163.com (Z.Y.); 2 Department of Natural Resources and Environmental Sciences, Alabama A&M University, Huntsville, AL 35801, USA; phtrangvn85@gmail.com (T.P.); xianyan.kuang@aamu.edu (X.K.)

**Keywords:** *Miscanthus sacchariflorus*, *M. lutarioriparius*, cold acclimation, relative electrical conductivity, LT_50_

## Abstract

We aim to provide a theoretical basis for improving the cold tolerance of *Miscanthus* spp., a widely recognized C4 perennial bioenergy crop, and extending its application in the industry. This study evaluated its cold tolerance by measuring the relative electrical conductivity (REC) of detached leaves. We calculated the half-lethal temperature (LT_50_) during non-acclimation and acclimation treatments in the 12 wild genotypes of *M. sacchariflorus* and *M. lutarioriparius* from different regions of China. In this study, five temperature treatments were carried out to simulate the natural early spring cold process, with temperatures of 0 °C, −4 °C, −8 °C, −12 °C, and−16 °C. We compared the REC and LT_50_ during the non-acclimation and acclimation treatments, and the results show that the REC of the 12 genotypes increased with a decrease in the treatment temperature, forming an S-shaped curve, which was significantly negatively correlated with the corresponding temperature. Under non-cold acclimation, the *M. sacchariflorus* B0111 from Jiamusi, Heilongjiang Province, had the lowest LT_50_ of −9.49 °C, showing extraordinarily strong cold tolerance. However, *M. lutarioriparius* A0630 from Shaoxing, Zhejiang Province, had the highest LT_50_ of −6.43 °C, demonstrating the weakest cold tolerance. After 21 days of cold acclimation, B0111 still exhibited the most substantial cold tolerance. While A0630 showed an enhanced cold tolerance, it remains the weakest in this study. The cold acclimation abilities of the 12 genotypes varied from −0.016 to 0.666 °C. Additionally, we found that the tolerance abilities of *Miscanthus* were enhanced after acclimation. Furthermore, its tolerance capacity was found to vary according to the geographic factor of its original location, which shows the significance of the correlation with latitude. However, there was no correlation found with altitude or longitude.

## 1. Introduction

*Miscanthus* spp., belonging to the Poaceae family, of the Andropogoneae tribe [1,2], is a kind of C4 perennial grass and a promising second-generation bioenergy crop [3,4,5,6,7,8,9,10]. Among the 14 species of *Miscanthus*, the four core species considered the most suitable as energy crops are *M. floridulus*, *M. sinensis*, *M. sacchariflorus*, and *M. lutarioriparius* [11,12,13]. Furthermore, a natural sterile triploid cultivar, *Miscanthus* × *giganteus* (M × *g*), crossed with tetraploid *M. sacchariflorus* and diploid *M. sinensis* in southern Japan, has played a particularly key role in the *Miscanthus* family, being used as bioenergy feedstock and in biobased products [14,15,16,17,18,19,20,21,22]. However, as a sterile genotype, M × *g* is severely limited by two major faults: (1) a significant lack of genetic diversity and (2) an insufficient overwintering ability in temperate regions with cold winters [23,24,25,26,27,28,29,30]. Improving its genetic diversity and developing its cold stress capacity are the current breeding goals for *Miscanthus* to meet cold tolerance breeding requirements. Therefore, understanding its cold tolerance ability and screening and evaluating *Miscanthus* germplasm are considered as essential for *Miscanthus* cold-tolerant breeding.

One of the original parents of M × *g*, *M. sacchariflorus* is a species of the *Miscanthus* genus which is distributed in the north and has the highest cold tolerance of all; thus, it is an attractive subject in the *Miscanthus* research field. To date, several studies have focused on this topic, including the screening and assessment of its cold tolerance, genetic diversity, and genetic population structure [31,32,33,34,35,36,37]. According to the current study, *M. sacchariflorus* can be found in 17 provinces across China, ranging from 30° N to 47° N, 106° E to 113° E, and 0 m to 1400 m. As a sibling species of *M. sacchariflorus*, *M. lutarioriparius* is a unique species in China. It is distributed across seven provinces, ranging from 28° to 34° N and 111° to 119° E at an altitude between 0 and 300 m [1,38]. It has been observed that *M. sacchariflorus* gradually evolves into *M. lutarioriparius* as it moves from high to low latitudes. Although *M. lutarioriparius* has been distinguished from *M. sacchariflorus* using morphological analyses, it cannot be distinguished at the molecular level by using markers such as SSR, ISSR, trnL-F, or rpl16 [39]. Considering the differences in morphology, distribution, habitat, and flowering time, it has been proposed that *M. lutarioriparius* is a variety of *M. sacchariflorus* [35]. Temperature is a critical factor influencing the distribution range of the two species. Understanding and investigating the cold tolerance of *M. sacchariflorus* and *M. lutarioriparius* is essential in the further genetic improvement and expansion of their cultivation range. Compared to the unstable and difficult-to-operate physiological indicators, measuring the relative electrical conductivity of plants in low-temperature environments and calculating their half-lethal temperature is a more effective method. This method involves measuring the electrical conductivity of plant tissues at a range of temperatures, fitting the data to a logistic regression model, and using the temperature at the inflection point to represent the half-lethal temperature. The conductivity method, which involves measuring the electrolyte efflux rate of plant cells and fitting it with the logistic regression, provides a quantitative indicator of cold tolerance, specifically of the half-lethal temperature (LT_50_), reflecting the lowest temperatures that the plants can endure; this is used to evaluate their cold tolerance. This method has been widely applied to assess the cold tolerance of plants such as pitaya [40], *Camellia sinensis* [41], wheat [42], apple dwarfing rootstock [43], garlic [44], *Arabidopsis* [45], potatoes [46], grape [47], and *Xanthoceras sorbifolia* [48]. In a study of the cold tolerance of *M. sacchariflorus* and *M. lutarioriparius*, Du et al. [49,50] evaluated the cold tolerance of the *Miscanthus* species under different temperature treatments, simulating low-temperature stress and measuring physiological and biochemical indicators, including malondialdehyde (MDA), superoxide dismutase (SOD), peroxidase (POD), and proline (PRO), to assess the cold tolerance of *Miscanthus*. The relative electrical conductivity was also used to fit and calculate the lethal temperature (LT_50_), allowing for a comparison of the cold tolerance of different *Miscanthus* genotypes from various collection sites in China. To date, few reports on the half-lethal temperature have studied the cold tolerance of *Miscanthus*.

This study assessed 12 *Miscanthus* genotypes and analyzed the variations in the relative electrical conductivity of the different genotypes under low-temperature stress by simulating a cold environment. By fitting the logistic regression and calculating the half-lethal temperature of these genotypes, this study evaluated their cold tolerance capabilities, providing a theoretical basis for screening and cultivating cold-resistant traits in *Miscanthus* species and laying the foundation for subsequent research into the cold tolerance of the *Miscanthus* germplasm.

## 2. Results

### 2.1. REC Variation Under Cold Non-Acclimation

**Figure 1:**
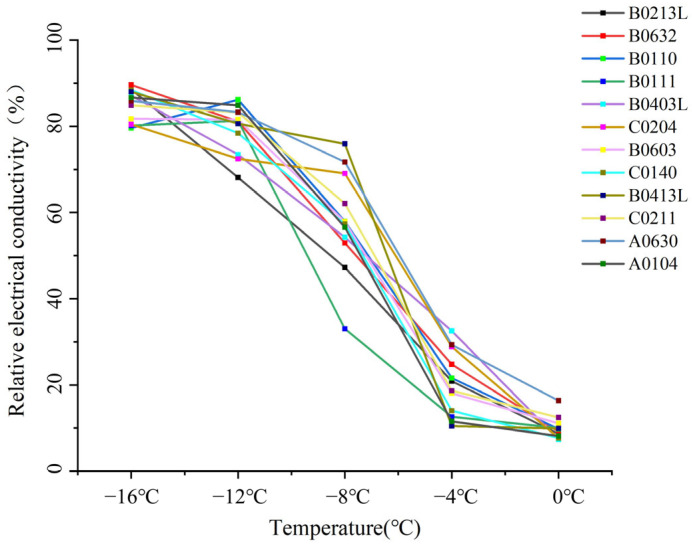
Trends in relative electrical conductivity (REC) among 12 *Miscanthus* genotypes under non-acclimated conditions. REC values were measured at five low-temperature levels (0 °C, –4 °C, –8 °C, –12 °C, –16 °C) for each genotype.

The extent of electrolyte efflux from plant cells can be represented by the relative electrical conductivity (REC), which reflects the degree of low-temperature damage to the plant cell membrane system [51]. Under non-cold-acclimation conditions, the changes in relative electrical conductivity (REC) of the leaves of the 12 genotypes under different temperature treatments are shown in Figure 1 and Table 1. The trend crosses five temperatures (0 °C, −4 °C, −8 °C, −12 °C, and −16 °C) for all 12 genotypes, forming an “S” curve. The results indicate that under the 0 °C treatment, the range of REC of the 12 genotypes was between 7.38% and 16.37%. At 0 °C, genotype A0630 exhibited the highest observed REC value (16.37%) among all genotypes, suggesting a potentially greater sensitivity to low temperature at this treatment level. Under low-temperature treatment, the upward trend in REC varied among different genotypes. B0403L showed the largest increase in REC between 0 °C and −4 °C, which was up to 25.18%. Nine tested genotypes exhibited the largest increase in REC between −4 °C and −8 °C, with B0413L showing a significant increase of 65.44% in this temperature range. Two tested genotypes showed the most significant rise in REC between −8 °C and −12 °C: B0111 and B0632. Within the temperature range of −12 to −16 °C, the overall trend of REC across all genotypes was a slow rise with slight changes. However, B0110 and B0111 had a negative increase in REC, meaning that the REC decreased by 6.58% and 1.14%, respectively.

### 2.2. REC Variation Under Cold Acclimation

**Figure 2:**
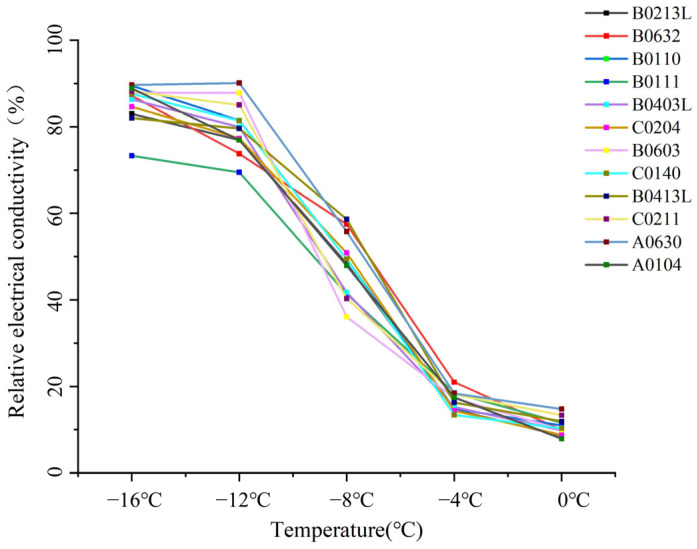
Trends in relative electrical conductivity (REC) among 12 *Miscanthus* genotypes under cold-acclimated conditions. REC values were measured at five low-temperature levels (0 °C, –4 °C, –8 °C, –12 °C, –16 °C) for each genotype.

The changes in REC of the 12 genotypes under different temperature treatments and after 21 days of cold acclimation are illustrated in Figure 2 and Table 2. The trends of the 12 genotypes still represent an “S” curve. Compared with non-cold-acclimated genotypes, the comparatively concentrated relative conductivity values of each genotype at the same temperature indicate that the cold adaptation capacities of different genotypes tended to converge after 21 days of cold acclimation. According to Table 2, the results suggest that under the 0 °C temperature treatment, the range of relative conductivity for the 12 genotypes was between 7.93% and 14.79%. At the temperature of −4 °C, the range was between 13.42% and 20.99%. Under the −8 °C temperature treatment, the range was between 36.08% and 58.63%, and under the −12 °C temperature treatment, it was between 69.49% and 90.14%. Under the −16 °C temperature treatment, the range was between 73.31% and 89.69%. Eight tested genotypes exhibited the largest increase in relative conductivity between −4 °C and −8 °C, with an increase ranging from 30.45 to 42.31%. Four tested genotypes showed the largest increase in relative conductivity between −8 °C and −12 °C, specifically, C0211, B0111, B0603, and B0403L, with B0603 showing the greatest increase (51.75%). Within the temperature range of −12 to −16 °C, the relative conductivity of ten genotypes slightly increased, but two genotypes showed a different trend from the others. A0630 experienced a negative increase, with a decrease of 0.45% in relative conductivity at −16 °C compared to −12 °C. B0603 did not demonstrate any difference in relative conductivity between −12 °C and −16 °C, maintaining the value of 87.83% under both temperature treatments.

### 2.3. LT_50_ Variation Under Non-Cold-Acclimated and Cold-Acclimated Tolerance

The logistic equations, correlation coefficients, and half-lethal temperatures of the 12 genotypes under non-cold acclimation and cold acclimation are presented in Table 3. It can be observed that under non-cold-acclimation conditions, the half-lethal temperature range of the 12 genotypes was from −6.43 to −9.49 °C, with most concentrated around the average value of −8.11 °C.

After 21 days (about 3 weeks) of cold acclimation, the REC of the test materials was immediately measured, and the LT_50_ was calculated by fitting the logistic regression. The results showed that the relationship curve between the REC and temperature of the tested genotypes could be well fitted by the logistic regression equation, with fitting values ranging from 0.9563 to 0.9963. It can be observed that the LT_50_ range of the 12 genotypes was from −7.05 to −9.93 °C, with the majority concentrated around the average value of 8.46 °C.

Comparison of the LT_50_ under non-cold and cold acclimation of the 12 genotypes reveals a range of −0.016 to 0.666 °C. Among the twelve genotypes, two have a cold acclimation ability of less than 0.1 °C, specifically B0603 and C0140, with C0140 exhibiting the lowest adaptability, as evidenced by an increase in its LT_50_ value of 0.016 following domestication. There are seven genotypes with an acclimation ability between 0.1 °C and 0.5 °C, which are B0213L, B0632, B0110, B0111, B0413L, C0211, and A0104, respectively. Three genotypes have an acclimation ability between 0.5 °C and 1.0 °C: B0403L, C0204, and A0630. Among the twelve genotypes examined, the C0204 genotype exhibited the highest level of cold tolerance, with a recorded value of 0.666 °C, indicating a robust capacity to endure cold temperatures. The variation in cold acclimation ability among the genotypes suggests that there are differences in their capacity to adjust to a low-temperature environment. Genotypes with stronger cold acclimation abilities may have more effective mechanisms for coping with cold stress, such as the production of protective molecules, changes in gene expression, or alterations in cellular metabolism that enhance their freezing tolerance.

### 2.4. The Correlation Between LT_50_ and Geographic Factors

**Figure 3:**
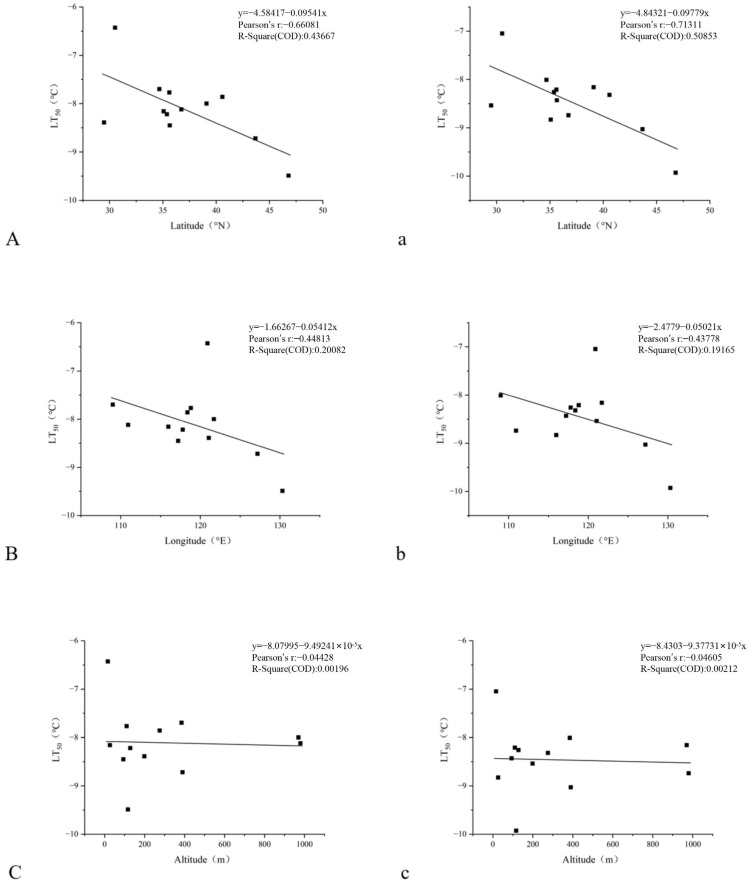
Linear regression equations of LT_50_ under non-cold and cold acclimation with latitude, longitude, and altitude. Note: (**A**–**C**) represent linear regression equations of LT_50_ under non-cold acclimation with latitude, longitude, and altitude, respectively; (**a**–**c**) represent linear regression equations of LT_50_ under cold acclimation with latitude, longitude, and altitude, respectively.

The correlation analysis results indicate significant relationships between the LT_50_ under non-cold acclimation and geographic factors. The correlation coefficient between the LT_50_ and latitude is −0.661, with a *p*-value of 0.019 (Figure 3A). This indicates a significant negative correlation at the 0.05 level. The negative correlation suggests that genotypes from higher latitudes (which typically have colder climates) tend to have lower LT_50_ values, meaning they have a higher cold tolerance and are better adapted to withstand low temperatures. The correlation coefficient between the LT_50_ and longitude is −0.448, with the *p*-value = 0.144 > 0.05, indicating no correlation between the LT_50_ and longitude (Figure 3B). The correlation coefficient between the LT_50_ and altitude is −0.044, close to zero, and the *p*-value is 0.891, greater than 0.05 (Figure 3C). This suggests that there is no significant correlation between the LT_50_ and altitude, indicating that the cold tolerance of the genotypes is not significantly influenced by the altitude of their origin.

With cold acclimation, the correlation analysis indicates significant relationships between the LT_50_ and geographic factors. The correlation coefficient between the LT_50_ and latitude is −0.713, with a *p*-value of 0.009 (Figure 3a). This suggests a significant negative correlation at the 0.01 level. Similar to the results before cold acclimation, this indicates that genotypes from higher latitudes, which tend to be colder, generally have lower LT_50_ values after cold acclimation, implying that they have improved their cold tolerance and are better prepared to withstand low temperatures. The correlation coefficient between the LT_50_ and longitude is −0.438, with a *p*-value of 0.155 (Figure 3b). This also indicates no correlation between the LT_50_ and longitude. The correlation coefficient between the LT_50_ and altitude is −0.046, close to zero, and the *p*-value is 0.887, greater than 0.05 (Figure 3c). This indicates no significant correlation between the LT_50_ and altitude after cold acclimation, suggesting that the cold tolerance of the genotypes is not significantly affected by the altitude of their origin, even after the acclimation process.

## 3. Discussion

### 3.1. REC in Cold Tolerance

When plants experience low temperatures, the selective permeability of the cell membrane is altered, leading to leakage of cellular electrolytes and an increase in relative electrical conductivity [52,53]. Therefore, the REC of a plant can reflect the degree of cell membrane damage, thereby indirectly reflecting the cold tolerance of the plant tissues. The REC demonstrates the rate of intracellular electrolyte leakage before and after cell membrane damage under low-temperature conditions. The more electrolytes leak out, the more the relative electrical conductivity indicates a higher degree of cell membrane damage and weaker cold tolerance. Conversely, a lower relative electrical conductivity indicates stronger cold tolerance. An increase in relative electrical conductivity more accurately reflects the degree of cell membrane damage and is a reliable indicator for evaluating the strength of a plant’s cold tolerance [40].

In the initial stages of low-temperature treatment, the change in relative electrical conductivity (REC) for all genotypes remains flat. This may be a protective mechanism of plants when subjected to adverse conditions, perhaps because the cell membrane is still in a reversible stage of damage at this time and capable of regular active transport, thereby adjusting the leakage of the cytoplasm and stabilizing its relative electrical conductivity [54]. In the later stages of low-temperature treatment, the REC of all genotypes exceeds 80%, indicating that the cell membrane has been severely damaged and is in an irreversible state, with impaired transport function.

### 3.2. LT_50_ in Cold Tolerance

Various factors influence plant cold tolerance, and the physiological processes related to cold tolerance are complex. Field assessment is widely regarded as the most reliable method. Still, it is influenced by the environment, requiring repeated trials over multiple years and locations, which are time-consuming and labor-intensive. Therefore, a rapid, accurate, and simple identification method is needed [55]. Using the relative electrical conductivity to fit the logistic equation to calculate the low-temperature half-lethal temperature of each genotype of *M. sacchariflorus* and *M. lutarioriparius* is simple, fast, and cost-effective. It can better determine their cold tolerance [56]. In this study, 12 genotypes of *Miscanthus* were tested, and their relative electrical conductivity changes followed the logistic equation curve pattern. The low-temperature LT_50_ of the 12 genotypes under non-cold and cold acclimation was −6.43 to −9.49 °C, and after cold acclimation, it was −7.05 to −9.93 °C, which is consistent with the LT_50_ range of wheat [55].

It is worth noting that cold stress in natural environments typically develops gradually, allowing plants to initiate various acclimation responses over time. These include membrane lipid remodeling, increased fatty acid unsaturation, and the accumulation of cryoprotective substances; these responses collectively enhance membrane stability and cold tolerance [57]. However, in this study, low-temperature stress was applied in a rapid and controlled manner to standardize experimental conditions and assess the innate cold tolerance of *Miscanthus* genotypes. Under such abrupt cold exposure, the opportunity for complete physiological acclimation is limited. Therefore, the measured REC and LT_50_ values, particularly under non-acclimated conditions, likely reflect inherent genotypic differences in basal cold tolerance rather than inducible responses. Recognizing this distinction enhances the interpretation of our results and underscores the importance of future experiments incorporating gradual cold stress to more closely replicate natural overwintering scenarios.

### 3.3. Geographical Factors Impact the Cold Tolerance

By analyzing the linear regression equations and the Pearson correlation coefficients, one can determine whether there is a significant correlation between the LT_50_ and the geographic factors, as well as the trend (positive or negative) of that correlation. A significant correlation would suggest that the geographical factors may play a role in the cold tolerance of the genotypes, which could help select genotypes for cultivation in specific geographical locations or in breeding programs aimed at improving cold tolerance.

Correlation analysis was performed between the LT_50_ under non-cold and cold acclimation, the cold acclimation ability of each genotype, and the latitude, longitude, and altitude of their collection sites. In this study, the analysis results indicate a highly significant negative correlation between the LT_50_ under non-cold acclimation and latitude, a significant negative correlation, and no correlation with longitude and altitude. Under cold acclimation, there is a significant negative correlation between LT_50_ and latitude and no correlation with longitude and altitude. Latitude is one of the crucial factors affecting the cold tolerance of *Miscanthus*. As the latitude increases, the cold tolerance of *M. sacchariflorus* and *M. lutarioriparius* increases, and the low-temperature half-lethal temperature will also decrease (and vice versa).

There are certain limitations to this experiment because only the relative electrical conductivity of seedling plant leaves was measured, and the impact of different growth periods on the half-lethal temperature of each *Miscanthus* genus group was not considered. Some researchers also believe that the determination of the half-lethal temperature is based on the results of simulated low-temperature environments and cannot fully reflect the freeze damage under natural conditions. The consistency of the test materials will also affect their accuracy [58]. Therefore, the determination of the LT_50_ of the leaves in this study is only one of the bases for comparing their cold tolerance. Suppose we want to judge the cold tolerance of each group more accurately. In that case, we also need to combine relevant physiological and biochemical indicators of cold tolerance, such as total chlorophyll content, malondialdehyde (MDA), peroxidase (POD), proline, superoxide dismutase (SOD), and catalase (CAT), for a comprehensive evaluation. If the indirect measurement results in the laboratory can be combined with field identification results to evaluate the cold tolerance of *Miscanthus* plants, it will have a better effect [59].

### 3.4. Cold Tolerance Acclimation Mechanism

In this study, these findings suggest that the response to low-temperature stress is genotype-dependent, with some genotypes showing a more pronounced increase in membrane damage as the temperature decreases. The negative increase in relative conductivity observed in B0110 and B0111 at the lowest temperatures might indicate a protective response or an adaptation mechanism that reduces membrane permeability under extreme cold stress. This could be a survival strategy to prevent excessive loss of cellular contents, which could lead to cell death. The variation in the response to temperature stress among genotypes highlights the genetic diversity in cold tolerance. It provides valuable information for breeding programs aimed at improving cold tolerance in *Miscanthus* species.

Additionally, these findings suggest that cold acclimation has a significant impact on the cold tolerance of different genotypes. The increase in relative conductivity at higher sub-zero temperatures (−4 to −8 °C) suggests that the initial cold stress causes a rapid increase in membrane permeability. The most significant increase in relative electrical conductivity for some genotypes between −8 °C and −12 °C indicates a critical temperature range where cell membranes are most susceptible to damage. The stabilization or slight increase in relative conductivity at the lowest temperatures (−12 to −16 °C) may reflect the plants’ adaptive mechanisms to extreme cold, such as the production of protective compounds or the ability to repair membrane damage. The genotypes that show no change or a decrease in relative conductivity at the lowest temperatures may possess more effective cold tolerance mechanisms, which could be a focus for future breeding efforts to improve cold tolerance in *Miscanthus* species.

### 3.5. Cold Tolerance Breeding in Miscanthus

As we know, plants with more northerly distributions that experience colder winter temperatures exhibit more cold tolerance and acclimation capacity than the more southerly species. In our study, climatic range estimates indicated that *M. sacchariflorus,* originally distributed in colder regions, has a colder tolerance capacity than *M. lutarioriparius*, which is found in warmer regions. Moreover, it was proved that the genotype collected from higher latitudes will have colder tolerance and cold acclimation adaptation than those from lower latitudes. In this study, the lowest LT_50_ was −9.49 °C (B0111 from Jiamusi, Heilongjiang province, the northeast province of China where the average temperature in January was −30.9 to −17.4 °C). In terms of *M. lutarioriparius*, the lowest LT_50_ was −6.43 °C (A0630 from Jiaxing, Zhejiang province, a southern province of China where the average temperature in January was −4 to −2 °C). Meanwhile, the comparison of the significantly lowest mean temperature of the natural distribution of two species, *M. sacchariflorus* and *M. lutarioriparius*, was −23.21 °C and −7.29 °C, respectively [38].

These findings are essential for understanding the geographical adaptation of *Miscanthus* genotypes and can inform breeding and cultivation strategies. Genotypes from higher latitudes and specific longitudes may be more suitable for cultivation in colder climates, while altitude has less influence on the cold tolerance of these plants. This information can be used to select appropriate genotypes for specific environments and to inform breeding programs aimed at improving the cold tolerance of *Miscanthus* species [60].

## 4. Materials and Methods

### 4.1. Plant Materials

The experimental materials were selected from the *Miscanthus* Germplasm Nursery in Changsha City, China (28.18° N, 113.07° E). The information on *Miscanthus* accessions is shown in Table 4.

### 4.2. Rhizome-Derived Propagation and Regrowth Management

In December 2021, we dug ~200 g of rhizomes of each of the studied genotypes from the field (*Miscanthus* germplasm nursery at Hunan Agricultural University). We propagated them in a tray with a mesh net (size: 30 × 30 × 5 cm). To prevent the rhizome from growing outside of the tray, we put a 30 × 30 cm non-woven fabric under it. Each genotype was hosted by a tray, and all trays were placed on a cultivation tank with substrate in the greenhouse. With two years of growth (year 2022 and 2023), all *Miscanthus* accessions had grown an adequate number of vigorous rhizomes in their tray. In February 2024, we trimmed the biomass above the soil, manually separated the rhizomes from the bottom of the tray, and washed the soil off the rhizomes thoroughly. The rhizomes were cut into ~3 cm long pieces with 2~3 new buds, and we transplanted the fragments into a 50-cell tray (size: 53 × 27 × 4 cm). Each cell hosted one piece. Then, we allowed the plants to establish themselves for one month in the greenhouse. To ensure uniform growth conditions, in April 2023, when the new shoots had grown to ~10 cm, the regenerated plants in the 50-cell tray were transferred to the growth room, which had a temperature of 25 °C, a relative humidity of 80%, and a 16-h day. During the preparation experiment, all accessions experienced another month to establish themselves in our growth room, and during this time, all rhizome-derived plants were irrigated at regular intervals, and 0.5× Hoagland’s nutrient solution was provided as needed to ensure optimal nutrient availability.

### 4.3. Low-Temperature Treatment

Low-temperature treatments were performed using an XMTF-7000 high-precision low-temperature test chamber (Cangzhou Xinxing Testing Instrument Co., Ltd., Cangzhou, China), which maintained temperature control with an accuracy of ±0.5 °C. The procedure was adapted from Zhang [61], with slight modifications to accommodate our specific experimental requirements. Mature leaves with uniform morphology and growth conditions were selected for analysis. First, leaf tips and veins were removed, and the remaining portions of the leaves were cut into approximately 1 cm × 1 cm squares. These segments were then placed into 10 mL centrifuge tubes containing 2 mL of deionized water. To ensure thorough contact between the leaf tissues and water, the tubes were shaken by hand several times. Subsequently, the samples in the centrifuge tubes were subjected to low-temperature treatment at −4 °C, −8 °C, −12 °C, and −16 °C for 12 h each.

### 4.4. Low-Temperature Acclimation

Plant materials with healthy leaves were subjected to low-temperature acclimation by placing them in a 4 °C refrigerator for 21 days. During this time, 0.5× Hoagland’s nutrient solution was provided weekly to support the necessary nutrition. The lighting was maintained at 16 h a day. Following the acclimation period, the relative electrical conductivity of the leaves was measured immediately to evaluate the plant cell damage caused by exposure to low temperatures.

### 4.5. Electrolyte Leakage Assessment

The measurement of relative electrical conductivity in detached leaves is an established method for assessing cold tolerance in plants, indicating cellular damage resulting from cold exposure through electrolyte leakage [40,48,51]. This leakage reflects the integrity of cell membranes, with increased conductivity indicating greater damage. First, the detached leaves were unfrozen and then placed on an orbital shaker; the shaker oscillated for 20 min. After reaching equilibrium, the initial electrical conductivity (R_1_) was measured using a conductivity meter. Subsequently, the samples were boiled for 10 min to release all possible electrolytes from the cells, cooled to room temperature (25 ± 1 °C), and then oscillated again for 20 min. After another equilibration period, the final conductivity (R_2_) was recorded. A control measurement (R_0_), without any plant samples, was also conducted to ensure that baseline conductivity levels were accounted for. Last, the relative electrical conductivity (REC) was calculated using the formula:


$$\mathrm{Relative Electrical Conductivity}\;(\mathrm{REC})=[(R_{1}-R_{0})/(R_{2}-R_{0})]\times 100\%$$

This calculation provides a percentage that quantifies the extent of cellular damage due to cold stress, with higher values indicating greater damage and lower values indicating cold tolerance.

### 4.6. Determination of Half-Lethal Temperature

The relative electrical conductivity obtained from each temperature treatment was analyzed using logistic regression to determine the plant’s cold tolerance. The model is represented by the equation: y = k/(1 + ae^−bx^), where *y* is the relative conductivity, x is the temperature of the treatment, k represents the saturation capacity of the cell damage rate (set to 100 in this experiment), and a and b are the curve-fitting parameters.

To determine parameters a and b, the logistic equation is transformed into a linear form by defining a new variable y′ as: y’ = ln[(k−y)/y]. This transformation converts the original logistic equation into ln[(k − y)/y] = ln(a) − bx, linking he transformed relative conductivity y′ linearly to the treatment temperature x. Linear regression is then applied to estimate the parameters *a* and *b* and the correlation coefficient r. The half-lethal temperature (LT_50_), indicating the point at which 50% of the cellular membranes are damaged, is calculated using the derived parameters from the regression: x = ln(a)/b. This LT_50_ value is critical in quantifying the plant’s tolerance to cold stress, representing the temperature at which cellular damage becomes extensive. Previous studies, such as those by Wang [62], have employed similar methodologies to assess plant cold tolerance.

### 4.7. Data Analysis

The original data were analyzed using the Excel software, and the correlation coefficients and logistic regression were calculated using IBM SPSS Statistics 27.0.0 (https://www.ibm.com/docs/en/spss-statistics/27.0.0, accessed on 8 September 2024). Graphs were plotted using Origin Pro 2024 (https://www.originlab.com/2024, accessed on 8 September 2024).

## 5. Conclusions

1. We found variability within and among species in cold tolerance. The 12 tested genotypes exhibited varying cold tolerance levels under the same treatment. In general, *M. sacchariflorus* was far more cold tolerant than *M. lutarioriparius.* These extremely cold-tolerant genotypes are excellent candidates for studying both the molecular and ecological aspects of cold tolerance.
2. Relative electrical conduction is a strategy for screening the cold tolerance in *Miscanthus*, and 21 days of cold acclimation is a suitable method for improving the capacity of cold tolerance in *Miscanthus*.
3. Whether it undergoes non-cold or cold acclimation, the cold tolerance of *Miscanthus* is significantly related to the geographical factors of its original site, which indicates that tolerance breeding should consider the resource of germplasm.
4. It is important to note that while cold acclimation ability is one aspect of a plant’s overall cold tolerance, it is also influenced by other factors such as the duration and intensity of cold exposure, the plant’s growth stage, and its genetic makeup. Further research into the genetic basis of cold acclimation and its interaction with environmental factors can aid in the development of *Miscanthus* cultivars with enhanced cold tolerance for diverse geographic regions.

## Figures and Tables

**Table 1:** Relative electrical conductivity (REC) of 12 *Miscanthus* genotypes at five low-temperature levels under non-acclimated conditions.

No.	Genotype	Relative Electrical Conductivity (%)				
		0 °C	−4 °C	−8 °C	−12 °C	−16 °C
1	B0213L	8.7 ± 0.95 d	20.86 ± 13.53 d	47.27 ± 9.53 c	68.12 ± 3.17 b	88.6 ± 0.91 a
2	B0632	9.2 ± 0.4 e	24.81 ± 7.09 d	52.94 ± 4.17 c	81.15 ± 3.33 b	89.63 ± 2.32 a
3	B0110	9.79 ± 3.13 c	21.61 ± 14.16 c	57.95 ± 18.54 b	86.18 ± 5.68 a	79.6 ± 11.08 ab
4	B0111	10.02 ± 1.44 b	12.61 ± 2.28 b	33.04 ± 29.8 b	81.31 ± 10.76 a	80.17 ± 4.81 a
5	B0403L	7.38 ± 0.06 e	32.56 ± 4.37 d	54.26 ± 3.56 c	73.41 ± 5.47 b	87.11 ± 2.68 a
6	C0204	7.72 ± 1.61 c	28.87 ± 11.07 b	69.05 ± 15.46 a	72.45 ± 1.1 a	80.45 ± 1.45 a
7	B0603	11.13 ± 3.68 c	18.06 ± 4.01 c	57.88 ± 5 b	81.59 ± 7.58 a	81.74 ± 2.24 a
8	C0140	7.73 ± 1.4 e	14.04 ± 1.09 d	57.31 ± 4.82 c	78.4 ± 3.49 b	88.42 ± 2.01 a
9	B0413L	9.92 ± 1.01 c	10.5 ± 0.32 c	75.94 ± 6.06 b	80.59 ± 1.45 b	88 ± 3.88 a
10	C0211	12.46 ± 0.92 c	18.66 ± 5.54 c	62.09 ± 14.54 b	83.24 ± 8.53 a	84.84 ± 0.21 a
11	A0630	16.37 ± 3.78 b	29.34 ± 15.63 b	71.72 ± 10 a	83.28 ± 2.71 a	85.87 ± 0.96 a
12	A0104	8.18 ± 0.66 c	11.56 ± 0.51 c	56.6 ± 14.87 b	84.86 ± 5.5 a	86.64 ± 2.73 a

Note: Different lowercase letters indicate statistically significant differences (*p* < 0.05) among temperature treatments within the same genotype based on one-way ANOVA followed by LSD multiple comparison tests.

**Table 2:** Relative electrical conductivity (REC) of 12 *Miscanthus* genotypes at five low-temperature levels under cold-acclimated conditions.

No.	Genotype	Relative Electrical Conductivity (%)				
		0 °C	−4 °C	−8 °C	−12 °C	−16 °C
1	B0213L	8.61 ± 0.98 c	14.6 ± 3.66 c	48.37 ± 12.83 b	76.94 ± 2.74 a	82.99 ± 6.76 a
2	B0632	9.97 ± 1.14 d	20.99 ± 3.05 d	57.48 ± 4.27 c	73.77 ± 13.89 b	87.15 ± 3.19 a
3	B0110	11.01 ± 2.7 c	14.84 ± 1.78 c	49.41 ± 13.5 b	81.48 ± 8.91 a	89.51 ± 6.22 a
4	B0111	11.62 ± 2.69 c	18.48 ± 3.57 c	41.38 ± 8.63 b	69.49 ± 2.1 a	73.31 ± 0.42 a
5	B0403L	9.86 ± 1.56 c	15.22 ± 2.46 bc	41.74 ± 31.72 b	79.96 ± 2.58 a	86.32 ± 8.23 a
6	C0204	8.8 ± 1.82 c	14.41 ± 0.75 c	50.87 ± 4.67 b	77.32 ± 8.38 a	84.64 ± 5.41 a
7	B0603	9.96 ± 0.63 c	17.31 ± 2.01 c	36.08 ± 9.44 b	87.83 ± 0.25 a	87.83 ± 2.02 a
8	C0140	10.29 ± 1.64 c	13.42 ± 1.52 c	49.39 ± 12.5 b	81.4 ± 5.4 a	87.6 ± 3.15 a
9	B0413L	11.93 ± 1.9 c	16.32 ± 4.64 c	58.63 ± 9.61 b	79.62 ± 3.66 a	82.01 ± 8.24 a
10	C0211	13.36 ± 0.53 d	18.16 ± 0.85 c	40.31 ± 2.75 b	85.04 ± 2.75 a	88.19 ± 2.12 a
11	A0630	14.79 ± 2.57 c	18.41 ± 2.26 c	55.79 ± 35.2 b	90.14 ± 10.17 a	89.69 ± 10.07 a
12	A0104	7.93 ± 1.72 c	17.48 ± 1.14 c	47.92 ± 19.81 b	76.93 ± 5.67 a	88.89 ± 5.93 a

Note: Different lowercase letters indicate statistically significant differences (*p* < 0.05) among temperature treatments within the same genotype based on one-way ANOVA followed by LSD multiple comparison tests.

**Table 3:** LT_50_ and fitted logistic equations of 12 *Miscanthus* germplasms before and after cold acclimation.

Treatment	No.	Genotype	Regression Equation	R^2^	LT_50_
Non-cold acclimation	1	B0213L	y = 100/(1 + 10.763 e^0.272x^)	0.9986 **	−8.72
	2	B0632	y = 100/(1 + 9.252 e^0.287x^)	0.9959 **	−7.77
	3	B0110	y = 100/(1 + 7.817 e^0.257x^)	0.9427 **	−8.00
	4	B0111	y = 100/(1 + 12.331 e^0.265x^)	0.9510 **	−9.49
	5	B0403L	y = 100/(1 + 8.647 e^0.266x^)	0.9850 **	−8.12
	6	C0204	y = 100/(1 + 7.183 e^0.242x^)	0.9409 **	−8.16
	7	B0603	y = 100/(1 + 8.067 e^0.254x^)	0.9616 **	−8.22
	8	C0140	y = 100/(1 + 12.941 e^0.303x^)	0.9828 **	−8.45
	9	B0413L	y = 100/(1 + 10.478 e^0.299x^)	0.9239 **	−7.86
	10	C0211	y = 100/(1 + 7.422 e^0.26x^)	0.9626 **	−7.70
	11	A0630	y = 100/(1 + 4.504 e^0.234x^)	0.9618 **	−6.43
	12	A0104	y = 100/(1 + 13.276 e^0.308x^)	0.9615 **	−8.39
Cold acclimation	1	B0213L	y = 100/(1 + 11.637 e^0.272x^)	0.9808 **	−9.03
	2	B0632	y = 100/(1 + 8.788 e^0.265x^)	0.9914 **	−8.21
	3	B0110	y = 100/(1 + 10.872 e^0.292x^)	0.9815 **	−8.16
	4	B0111	y = 100/(1 + 8.024 e^0.21x^)	0.9804 **	−9.93
	5	B0403L	y = 100/(1 + 11.585 e^0.28x^)	0.9814 **	−8.74
	6	C0204	y = 100/(1 + 11.586 e^0.277x^)	0.9811 **	−8.83
	7	B0603	y = 100/(1 + 11.658 e^0.297x^)	0.9585 **	−8.26
	8	C0140	y = 100/(1 + 11.485 e^0.29x^)	0.9757 **	−8.43
	9	B0413L	y = 100/(1 + 8.06 e^0.251x^)	0.9600 **	−8.32
	10	C0211	y = 100/(1 + 9.066 e^0.275x^)	0.9658 **	−8.01
	11	A0630	y = 100/(1 + 7.632 e^0.288x^)	0.9563 **	−7.05
	12	A0104	y = 100/(1 + 12.484 e^0.295x^)	0.9963 **	−8.54

Note: ** represent fitting degrees that are remarkably significant at *p* < 0.01. LT_50_ (temperature at which 50% relative electrical conductivity occurs) was estimated for each genotype under non-acclimated and cold-acclimated conditions using a logistic regression model. Fitted equations were based on relative electrical conductivity (REC) data measured at five low temperatures (0 °C, –4 °C, –8 °C, –12 °C, –16 °C). Differences in LT_50_ values indicate changes in cold tolerance following acclimation.

**Table 4:** Information on plant materials used in this study.

No.	Genotype	Species	Collection Location	Latitude (°N)	Longitude (°E)	Altitude (m)
1	B0213L	*M. sacchariflorus*	Jiaohe, Jilin	43.683	127.183	390.00
2	B0632	*M. sacchariflorus*	Rizhao, Shandong	35.617	118.813	110.60
3	B0110	*M. sacchariflorus*	Dalian, Liaoning	39.100	121.719	970.00
4	B0111	*M. sacchariflorus*	Jiamusi, Heilongjiang	46.800	130.317	117.00
5	B0403L	*M. sacchariflorus*	Sixian, Shanxi	36.738	110.950	980.00
6	C0204	*M. sacchariflorus*	Chengwu, Shandong	35.081	115.994	26.93
7	B0603	*M. sacchariflorus*	Linyi, Shandong	35.384	117.803	128.41
8	C0140	*M. sacchariflorus*	Jining, Shandong	35.657	117.231	94.35
9	B0413L	*M. sacchariflorus*	Kuancheng, Hebei	40.593	118.396	276.00
10	C0211	*M. sacchariflorus*	Xianyang, Shaanxi	34.678	109.024	384.99
11	A0630	*M. lutarioriparius*	Jiaxing, Zhejiang	30.527	120.908	16.34
12	A0104	*M. lutarioriparius*	Shaoxing, Zhejiang	29.488	121.065	199.38

## Data Availability

The data are available from the corresponding author upon reasonable request.
